# Temporal and Spectral Optimization of Vegetation Indices for Estimating Grain Nitrogen Uptake and Late-Seasonal Nitrogen Traits in Wheat

**DOI:** 10.3390/s19214640

**Published:** 2019-10-25

**Authors:** Lukas Prey, Urs Schmidhalter

**Affiliations:** Chair of Plant Nutrition, Technical University of Munich, 85354 Freising, Germany; schmidhalter@wzw.tum.de

**Keywords:** high-throughput phenotyping, hyperspectral sensing, nitrogen uptake efficiency, phenomics, precision farming, proximal sensing, fungicide, red edge, remote sensing, sowing date

## Abstract

Grain nitrogen (N) uptake (GNup) in winter wheat (*Triticum aestivum* L.) is influenced by multiple components at the plant organ level and by pre- and post-flowering N uptake (Nup). Although spectral proximal high-throughput sensing is promising for field phenotyping, it was rarely evaluated for such N traits. Hence, 48 spectral vegetation indices (SVIs) were evaluated on 10 measurement days for the estimation of 34 N traits in four data subsets, representing the variation generated by six high-yielding cultivars, two N fertilization levels (N), two sowing dates (SD), and two fungicide (F) intensities. Close linear relationships (*p* < 0.001) were found for GNup both in response to cultivar differences (Cv; R^2^ = 0.52) and other agronomic treatments (R^2^ = 0.67 for Cv*F*N, R^2^ = 0.53 for Cv*SD*N and R^2^ = 0.57 for the combined treatments), notably during milk ripeness. Especially near-infrared (NIR)/red edge SVIs, such as the NDRE_770_750, outperformed NIR/visible light (VIS) indices. Index rankings and seasonal R^2^ values were similar for total Nup, while the N harvest index, which expresses the partitioning to the grain, was moderately estimated only during dough ripeness, primarily from indices detecting contrasting senescence between different fungicide intensities. Senescence-sensitive indices, including R787_R765 and TRCARI_OSAVI, performed best for N translocation efficiency and some organ-level N traits at maturity. Even though grain N concentration was best assessed by the red edge inflection point (REIP), the blue/green index (BGI) was more suited for leaf-level N traits at anthesis. When SVIs were quantitatively ranked by data subsets, a better agreement was found for GNup, total Nup, and grain N concentration than for several contributing N traits. The results suggest (i) a good general potential for estimating GNup and total Nup by (ii) red edge indices best used (iii) during milk and early dough ripeness. The estimation of contributing N traits differs according to the agronomic treatment.

## 1. Introduction

Although plant breeding in winter wheat traditionally focuses on the maximization of grain yield (GY) at sufficient grain N concentration (GNC), the efficient use of nitrogen (N) for grain N uptake (GNup) is gaining more attention for reducing N surplus and increasing the effective N uptake efficiency (NupEff) of a cropping system [1,2]. Similar to GY, GNup strongly depends on the environment. Moreover, interactions among agronomic treatment factors such as sowing date, fungicide intensity, N fertilization, and genotypes are relevant [3,4,5,6,7,8,9,10,11,12]. Expensive field trials have to be conducted for assessing the optimum treatment levels in different cropping environments, both for the selection of N-efficient genotypes in plant breeding, and for optimizing agronomic treatment factors [13].

However, being costly and time-consuming, the determination of plant traits is often restricted to only a few traits, such as GY and yield components, or it relies on visual scoring, which is subjective and expensive [14]. Non-destructive high-throughput proximal sensing has been developed for optimizing N management in the vegetative phase, and has become an approved tool in precision farming [15]. However, the generative phase is also important for maximizing GNup [16]. Post-anthesis N uptake (PANup) and N translocation (NT) respond to agronomic treatments, differ among cultivars, and influence total N uptake (Nup) as well as its partitioning to the grain [12,17]. Although vegetative Nup is primarily allocated to leaves, the organs which are more visible to spectral sensors [18], substantial amounts of N are allocated to stems and spikes until anthesis [19], and are predominantly translocated to the grain during the influence of senescence [20,21].

The spectral in-season estimation of GY has been addressed as a tool in plant phenotyping in various studies [22,23,24,25]. However, the estimation of GNup was evaluated less frequently, not to mention the contributing traits at the organ-level or seasonal dynamics. Compared from simulated band configuration, GNup was generally well estimated from red edge (RE) indices at milk ripeness, but also stem elongation was well suited in the presence of different N fertilization levels [26]. For breeding lines, Pavuluri et al. [27] reported better estimations of GNup and NupEff than those of GY, and better estimations for multi-year data than those for individual years. Moreover, correlations were closer under reduced N fertilization, ascribed to less saturation in thinner canopies. Using RE-based spectral vegetation indices (SVIs) in winter wheat, Frels et al. [28] complemented the estimation of GNup with that of PANup, total Nup, and the N harvest index (NHI). The estimation of PANup and NHI differed more between years than that of NupEff and GNup. They recommended the RE Maccioni index and identified the early grain filling stage as the most promising. Pavuluri et al. [27] suggested the related simple ratio index R780/R740 [29] and recommended the heading stage, followed by booting, stem elongation, and grain filling. For organ-level Nup in spring barley, Barmeier and Schmidhalter [18] reported better correlations for leaf blades and culms than for spikes and leaf sheaths.

Nonetheless, knowledge on the use of spectral methods for GNup and its contributing traits remains scarce, notably concerning high-yielding European conditions, as well as the impact of different agronomic treatments, compared to the use for estimating genotypic differences. Therefore, this study assesses the spectral estimation of the N traits previously compared in [12]. These traits include plant- and organ-level N concentration (NC) and Nup and traits related to NT and N allocation as a function of two levels of N fertilization, fungicide intensity and sowing dates for six winter wheat cultivars. We evaluated the (i) estimation potential by trait, (ii) identified suitable vegetation indices and (iii) optimum growth stages, and (iv) compared the differences in (i–iii) by underlying treatments. Although based on hyperspectral phenotyping, the results could be transferred to the use of simpler multispectral methods.

## 2. Materials and Methods

### 2.1. Experiment and Plant Sampling

The field trial, conducted as a split-split-plot design, comprised control (Cont)/reduced fungicide (RF)/early sowing (sowing date 1: SD1) on the main plot (MP), N level (N) on the sub-plot (SP), and cultivar (Cv) on the sub-sub-plot. It was conducted during the 2016/2017 growing season in southeastern Germany (48.406° N, 11.692° E) on homogeneous Cambisol of silty clay loam. The average annual temperature is approximately 8 °C and the annual precipitation approximately 800 mm. The previous crop grass-clover resulted in high soil mineral N content of 83 and 92 kg N ha^−1^ in SD1 and 69 and 87 kg N ha^−1^ in Cont in autumn and after winter, respectively. Six morphologically and phenologically differing cultivars (cv. Impression, JB Asano, Kerubino, Hybred, Hyland, Hystar) of winter wheat (*Triticum aestivum* L.) were drilled at 350 kernels m^−^^2^ in plots of 1.5 m in width and 6.4 m in length. Cultivars differed in anthesis date by up to six days, in plant height by 11 cm, in average plant density from 571–728 spikes per m^2^, and reacted differently to the agronomic treatments [12]. The sowing date was September 28 for SD1 and October 23 for Cont and RF. Within each MP, each cultivar was treated in two N levels, with N1 corresponding to 60 kg N ha^−1^ and N2 to 120 kg N ha^−1^, applied as ammonium nitrate in two equally split doses at tillering and stem elongation, respectively. The trial comprised four replicates for each factor combination (MP*N*Cv). Foliar fungicide was applied twice in Cont and SD1 plots, but not in RF plots. Fungicide was effective against the dominant pathogens *Erysiphe graminis*, *Septoria tritici,* and *Puccinia striiformis*, which showed only moderate incident in RF due to favorable weather conditions [12]. Above-average temperature and global radiation in March, May, and June together with high soil N supply resulted in strong vegetative growth, whereas below-average precipitation in May and June resulted in mild drought stress and accelerated senescence in some cultivars. Refer to [12] for details on the field trials, weather conditions, and treatment effects on the plant traits.

Biomass was sampled at anthesis (Zadok’s growth stage 65) and at physiological maturity (growth stage 95). For each genotype within each subplot, sampling dates were determined by visual scoring. In total 30 and 60 randomly selected shoots were cut per plot at the stem base at anthesis and maturity, respectively. Plant samples were manually separated into flag leaves, flag leaves–1 (second leaf from above), ‘other leaves’ (remaining leaves), culms including all leaf sheaths, and spikes. Plant samples were oven-dried at 50 °C for 2–4 days until no further weight loss was observed for subsequent determination of dry matter (DM) weight. After plant sampling at maturity, all plots were harvested (1–2 August 2017) using a combine harvester, to determine moisture-corrected grain yield (0%) for each plot. After grinding, the N concentration (NC) was determined by near-infrared spectroscopy (NIRS) using a FOSS NIRS 6500 (Foss, Silver Spring, MD, USA) and a Fourier-Transform -NIRS (Bruker, Billerica, MA, USA), whereas grains were analyzed as complete kernels. The calculation of ‘derived’ plant traits [20] comprised the N harvest index (NHI), post-anthesis N uptake (PANup), N translocation efficiency (NTEff), N translocation (NT), the contribution of PANup to total Nup (CPostNup), and the N uptake efficiency (NupEff), both at anthesis and at maturity. See [12] for details and Table S1 for a list of included traits.

### 2.2. Spectral Measurements and Data Preparation

**Figure 1:**
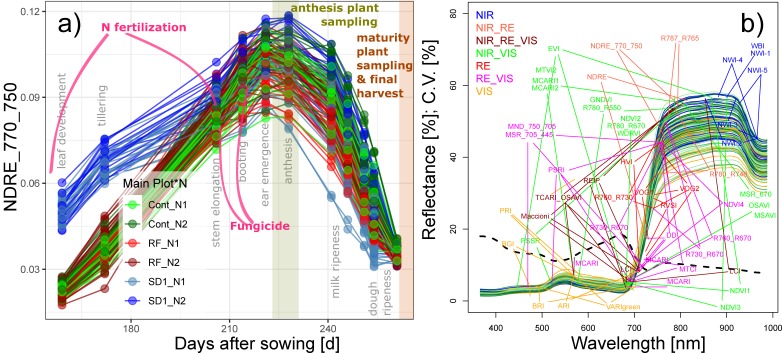
(**a**) Seasonal index development of the NDRE_770_750 colored by the main plot (Cont: Control; RF: reduced fungicide; SD1: sowing date 1) and N fertilization treatments (N1; N2). The last measurement date was not included in the analysis due to advanced senescence. Gray labels indicate growth stages; colored background bars indicate the reference plant sampling at anthesis and maturity. Days after sowing refer to the Cont and RF plots. (**b**) Plot-level spectra acquired on 21 June 2017 with band position of the tested spectral vegetation indices, colored by index groups: near-infrared (NIR: >765 nm), visible (VIS: <700 nm), and the extended red edge (RE: 700–765 nm). The spectra are colored by the values of grain nitrogen uptake (yellow: low, blue: high). The dashed line represents the coefficient of variation of the reflectance. Refer to Figure S2 for spectra of selected dates.

Spectral measurements were performed on 10 measurement days, covering all main growth stages from leaf development at the end of March until soft dough ripeness in July (Figure 1a; Table 1), using the mobile sensor platform PhenoTrac 4 [18]. It was equipped with a hyperspectral bidirectional passive point sensor spectrometer (tec5, Oberursel, Germany), measuring at a nominal resolution of 3.3 nm between 300 and 1000 nm at a frequency of 5 Hz. Plot boundaries were excluded and the distance to the canopy was ≈80 cm. The driving speed was ≈5 km h^−1^, resulting in a sampling distance of ≈28 cm at the top of the canopy and typically 15–20 registered spectra per target plot area, which allowed gapless sampling in a longitudinal direction in the middle of the plots. The resulting measurement strip was approximately 30–35 cm wide. Measurements were localized via a real-time kinematic global positioning system (RTK-GPS; Trimble, Sunnyvale, CA, USA). Based on polygons drawn to the core target plot area, the spectra were averaged to the plot level using custom-made LabView applications. Refer to Figure S1 for an example of the trial measurements. Spectra were smoothed using a 5 band moving average filter [30] for removing spectral noise. A total of 48 spectral vegetation indices (SVI) were calculated based on a literature review of related previous studies (Table 1). The SVIs were grouped by the included spectral range (visible light (VIS), red edge (RE), and near-infrared (NIR)), with VIS < 700 nm, ‘extended’ RE 700–765 nm, and NIR > 765 nm (Table 2; Figure 1b). In order to include SVIs with a NIR or RE band closer to the RE than that of the normalized difference vegetation index (NDVI; NIR = 780 nm), the upper boundary of the RE was higher than the common definition since the reflection still increased in region until approximately 770 nm.

### 2.3. Statistical Analysis

**Figure 2:**
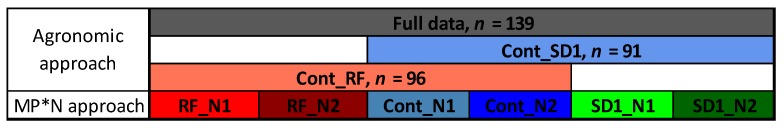
Datasets used for evaluating trait–index relationships. Three ‘agronomic’ datasets correspond to data of the whole trial (‘full data’), the combined main plots (MP) control (Cont) and reduced fungicide (RF; Cont_RF), as well as the combined MPs Cont and sowing date 1 (SD1; Cont_SD1). MP*N represents the testing within the six subplots as the interaction of main plots (MP) and N fertilization level (N; N1: 60 kg N ha^−1^; N2: 60 kg N ha^−1^). In SD1, five plots were removed due to missing data or plot damage.

SVIs were tested in simple linear regression analysis with the N traits for each sampling date, using the lm-function in R 3.4 (R Core Team, 2017). Relationships were compared by coefficient of determination (R^2^) and the root mean square error (RMSE) derived from the deviations between fitted and measured values. The relationships were compared for (i) the full data, (ii) across the combined Cont and SD1 data (Cont_SD1), (iii) across the combined Cont and RF data (Cont_RF), and (iv) within the six main plot*N level (MP*N) combinations (Figure 2) to assess the influence of the underlying treatments on the trait estimation. The MP*N blocks were considered as possible trial environments. Therefore, the results of these blocks were averaged and compared for the aggregated, averaged results. This approach can simulate conditions for phenotyping genotypic variation as relevant for plant breeding, whereas (i–iii) are referred to as ‘agronomic’ conditions. The relationships were compared with respect to estimation potential by trait, index ranking, and optimum measurement stages.

The indices were quantitatively ranked by their normalized performance for each trait in each dataset in order to overcome the influence of differing growing conditions as well as the date-specific index ranking. Therefore, within each of the three ‘agronomic’ datasets and the MP*N data, the across-date mean and maximum coefficients of determination (R^2^) of each index/trait combination were normalized to the trait-specific average mean and maximum R^2^ from all SVIs. In order to achieve a more robust ranking across underlying treatments, both the within-dataset mean and maximum rankings were summed up across the three ‘agronomic’ datasets. Consequently, the mean- and maximum-based rankings were combined through summing up both rankings (i) for the agronomic approach and (ii) for the MP*N approach. Considering a selection of indices robust towards date-specific suitability as more important, the mean-based ranking was double-weighted. These weighted mean/maximum-rank sums (WMMRS) were compared between the agronomic and the MP*N approach. WMMRS values were used for identifying one trait-specific optimum index from the ‘agronomic’ approach. A WMMRS < 9 indicates below-average and WMMRS > 9 indicates above-average index performance for a specific trait. The performance of this WMMRS-based best indices was compared over time both in the agronomic and the MP*N datasets. Additionally, the ‘agronomic’ rankings were compared to the WMMRS rankings of the MP*N approach by Spearman’s rank correlation coefficient.

In order to validate the initial index grouping based on the included spectral bands, the indices were grouped by a cluster analysis (Ward’s method) based on the coefficients of determination achieved for the studied traits.

## 3. Results

### 3.1. Optimized Date Selection by Plant Trait and Index

**Figure 3:**
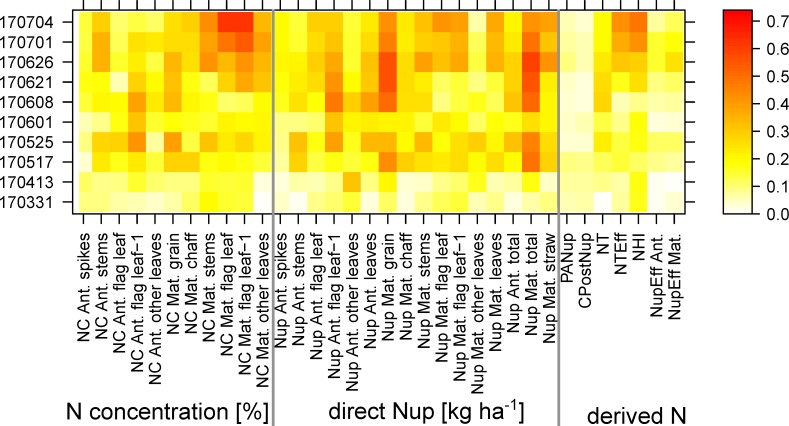
The influence of the measurement date (year/month/day) on the trait estimation in the full data: Maximum coefficients of determination (R^2^) found from the 48 tested spectral vegetation indices (SVIs). See Figure S3 for results of Cont_SD1, Cont_RF, and MP*N datasets.

The maximum coefficients of determination (R^2^) were calculated for each trait*date combination from all SVIs as a measure of the potential to estimate the 34 plant traits (Figure 3 for full data; Figure S3 for other datasets). The relationships were low or moderate (R^2^ < 0.40) before anthesis (06/08) but increased during grain filling. For most traits, relationships increased at stem elongation and booting (05/17 and 05/25, R^2^ ≈ 0.20–0.50), but decreased sharply around ear emergence (06/01). Low relationships were found for post-anthesis Nup (PANup), the contribution of PANup to total Nup (CPostNup), and Nup efficiency (NupEff). Regarding the N concentration (NC) traits, NC of flag leaves and flag leaves–1 at maturity were best assessed, particularly on the late days (beginning of July, dough ripeness). Among direct Nup traits, relationships were closest for grain Nup (GNup) and total Nup. Similar seasonal maximum R^2^ patterns were found for the other ‘agronomic’ datasets, Cont_SD1 and Cont_RF (Figure S3). For several traits, higher R^2^ values were found in Cont_SD1 during stem elongation (05/17 and 05/25) and during grain filling, as well as for various traits in MP*N.

### 3.2. Optimized Index and Date Selection Considering the UnderlyingTtreatments

#### 3.2.1. Grain Nup

**Figure 4:**
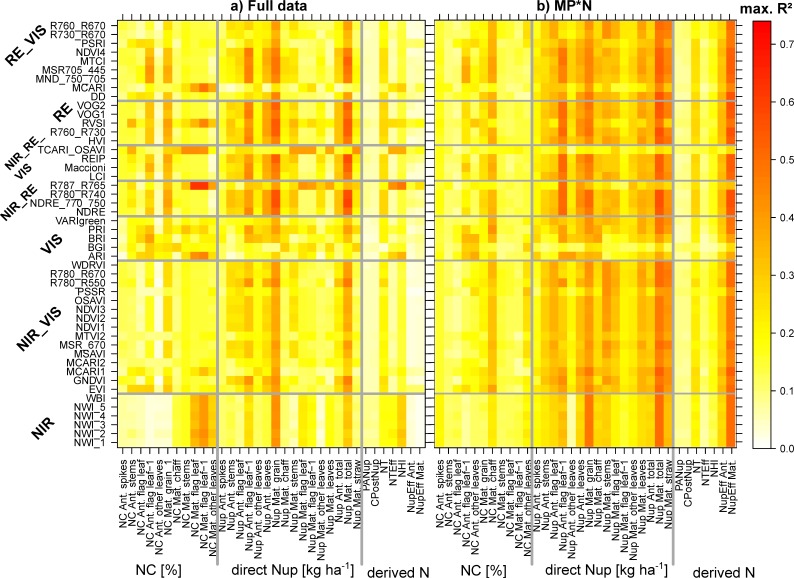
The index suitability by target trait: (**a**) full data; (**b**) average from the six MP*N datasets. Maximum coefficients of determination (R^2^) found for each index*trait combination from 10 measurement dates. Gray lines delimit index and trait groups. See Figure S4 for results of Cont_SD1 and Cont_RF.

**Figure 5:**
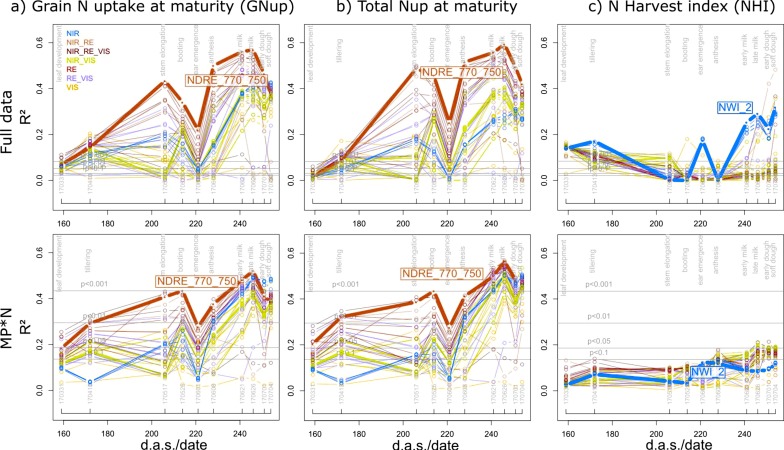
Seasonal coefficients of determination (R^2^) reached from all indices for (**a**) grain N uptake (GNup); (**b**) total Nup at maturity; and (**c**) the N harvest index (NHI) in the full data and the main plot*N (MP*N) approach. Lines are colored according to the spectral regions included in the index equations (Figure 1b). The results for the MP*N combinations are averaged from the results within the six MP*N combinations. Horizontal gray lines indicate significance thresholds on different levels. Thick lines and index labels indicate results of the best indices based on the WMMRS-ranking of the ‘agronomic’ approach. Refer to Figure S5 for results of the Cont_SD1 and Cont_RF datasets. *d.a.s.*: days after sowing.

**Figure 6:**
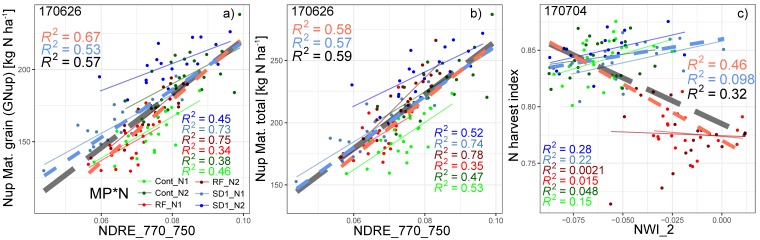
Relationships of (**a**) GNup, (**b**) total Nup and (**c**) N harvest index on most suited dates (year/month/day; selected based on Table 3) with rank-based best indices. Colored thin lines correspond to linear regressions for the MP*N subsets, dashed blue and red lines correspond to Cont_SD1 and Cont_RF, respectively, and the dashed gray lines indicate the full dataset. R^2^ values are colored accordingly.

The estimation of GNup primarily relied on the assessment of its ‘accumulative component’ (i.e., total Nup at maturity), which was well assessed in all datasets with the best index based on the weighted mean/maximum-rank sums (WMMRS), NDRE_750_770, always during milk ripeness (06/26; R^2^ = 0.57–0.59 in the agronomic datasets and R^2^ = 0.35–0.78 in the individual MP*N datasets; Table 3). In contrast, moderate relationships were observed for the ‘partitioning component’, N harvest index (NHI), only for the full data, Cont_RF and some MP*N combinations (Figure 4; Table 3). The index rankings were similar for GNup and total Nup with NIR/RE and NIR/RE/VIS combinations ranking highest both in the agronomic and MP*N datasets (Figure 5). GNup was similarly detected seasonally as total Nup (Figure 5a,b), with similar R^2^ values and the highest precision observed as well as milk ripeness (RMSE = 9.7–16.7 kg N ha^−1^; Table 3; Figure 6). In contrast, the water band index NWI_2 ranked highest for NHI; however, the relationships were not consistent for the different datasets (Figure 5c; Figure 6c). Weaker relationships were found for total Nup and GNup at booting/grain filling than at stem elongation, and especially during grain filling.

#### 3.2.2. Further Direct N traits

**Figure 7:**
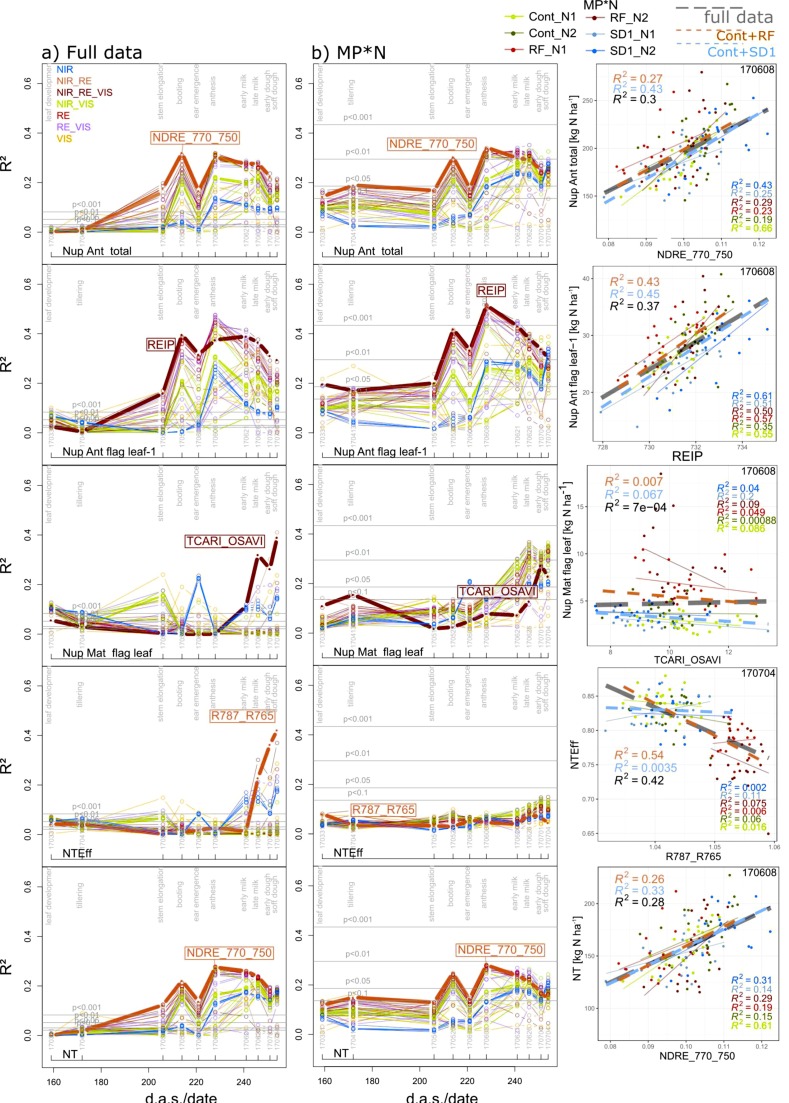
Seasonal coefficients of determination (R^2^) reached from all indices for selected N traits in (**a**) ther full data and in (**b**) the main plot*N (MP*N) data, as well as the trait–index relationships from the WMMRS-based best index/date (year/month/day) combinations. Lines are colored according to the spectral regions included in the index equations (Figure 1b). The results for MP*N are averaged from the results within the six MP*N combinations. Horizontal gray lines indicate significance thresholds on different levels. Thick lines and index labels indicate results of the best indices based on the WMMRS-ranking of the ‘agronomic’ approach. For the scatterplots, colored thin lines correspond to linear regressions for the MP*N subsets, dashed blue and red lines correspond to Cont_SD1 and Cont_RF, respectively, and the dashed gray lines indicate the full datasets. R^2^ values are colored accordingly. *d.a.s.*: days after sowing.

Index relationships for total Nup were weaker for anthesis Nup (Figure 7) than those for maturity Nup (Figure 5), but the index rankings were comparable. Moreover, the seasonal pattern was comparable for the full data, MP*N (Figure 7), and the other subsets (Figure S5 for total Nup). Both in the ‘agronomic’ and MP*N rankings, the index rankings for organ-level Nup were comparable with those for total Nup at anthesis and maturity (Figure 9). The Nup of spikes (at anthesis) and chaff (at maturity) was moderately detected by the WMMRS-based indices of the agronomic approach (R^2^ = 0.28–0.36) in Cont_SD1 and MP*N treatments (Table 3). Among the anthesis Nup traits, the Nup of flag leaves–1 was best detected (R^2^ from the REIP = 0.40–0.51 at anthesis; Table 3; Figure 7), followed by the Nup of flag leaves and stems (Table 3). In contrast, in spite of the good assessment of total Nup at maturity, only moderate relationships were found for straw Nup, primarily in the full data and in Cont_RF (R^2^ = 0.42 and 0.51, respectively), whereas the TCARI_OSAVI, the WMMRS-based best index in the agronomic approach, failed in the MP*N dataset (Table 3). The same index ranked highest for maturity Nup of stems, flag leaves, flag leaves–1, and the aggregated leaf Nup of all leaves, with moderate relationships (R^2^ = 0.37–0.46) in the full data and Cont_RF but weaker relationships in the MP*N data despite useful estimation of these traits from other indices (Figure 4).

#### 3.2.3. Derived N Traits

Although the post-anthesis component PANup was not sufficiently assessed by any index, moderate relationships (R^2^ = 0.26–0.33; Table 3) were found for the N translocation (NT) from the same index which ranked highest for total Nup at anthesis, NDRE_770_750, always at anthesis (Figure 7). In contrast, good relationships were observed from a few indices including the WMMRS-index R787_765 at soft dough ripeness for N translocation efficiency (NTEff) only in the full data (R^2^ = 0.42) and Cont_RF (R^2^ = 0.54) (Table 3; Figure 7)—driven mainly by the lower NTEff in the RF plots compared with the Cont and SD1 plots. For the NupEff at maturity, which relates the Nup to the amount of fertilized N, the WMMRS-based best index R787_765 primarily captured the influence of both N levels irrespective of the main plot treatments in the agronomic datasets (Table 3).

#### 3.2.4. N Concentration Traits

**Figure 8:**
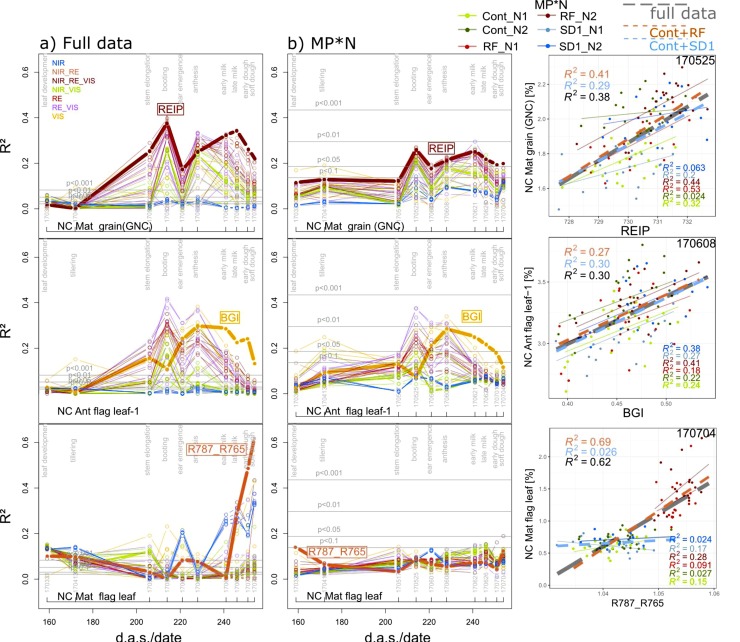
Seasonal coefficients of determination (R^2^) reached from all indices for selected N concentration (NC) traits in (**a**) the full data and in (**b**) the main plot*N (MP*N) data as well as the trait–index relationships from the best index/date (year/month/day) combination. Lines are colored according to the spectral regions included in the index equations (Figure 1b). The results for MP*N are averaged from the results within the six MP*N combinations. Horizontal gray lines indicate significance thresholds on different levels. Thick lines and index labels indicate results of the best indices based on the WMMRS-ranking of the ‘agronomic’ approach. For the scatterplots, colored thin lines correspond to linear regressions for the MP*N subsets, dashed blue and red lines correspond to Cont_SD1 and Cont_RF, respectively, and the dashed gray lines indicate the full datasets. R^2^ values are colored accordingly. *d.a.s.*: days after sowing.

Organ-level N concentration (NC) was rarely detectable in the MP*N subsets with the exceptions of flag leaves–1, and ‘other leaves’ at anthesis (06/08; R^2^ = 0.28 and R^2^ = 0.29 from the BGI), and grain NC (GNC; 05/25; R^2^ = 0.26 from the REIP; Table 3). Driven by the positive effect of N fertilization on GNC in all main plots, relationships for GNC were closer in the agronomic datasets than in most MP*N except the RF-MPs (Table 3; Figure 8), with R^2^ peaks of the WMMRS-index REIP at booting and milk ripeness. Since reduced fungicide use in RF affected the release of leaf nitrogen, leaf NC at maturity was well detected in the full data and Cont_RF. However, this was based on few indices, including the R787_765 and the MCARI, and restricted to late growth stages.

### 3.3. Index Rankings According to Trait and Dataset

**Figure 9:**
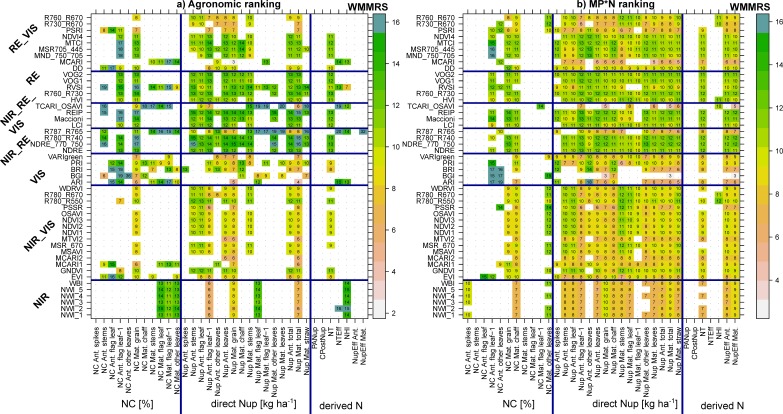
Weighted mean/maximum rank sums (WMMRS) for all evaluated trait*index combinations. (**a**) The combined WMMRS rankings from the three agronomic datasets (‘full data’, Cont_SD1, and Cont_RF). (**b**) WMMRS rankings from the six MP*N subsets, which were multiplied by three for direct comparison on the same numeric level. Rankings based on mean R^2^ across dates were double-weighted. For each trait, the value of nine corresponds to the average ranking across all indices. Blue cells indicate WMMRS values >15. Blue lines delimit index and trait groups, respectively. Rankings for trait*index combinations that did not exceed the threshold of maximum R^2^ values of 0.20 are not shown (white cells), being considered irrelevant.

The WMMRS are depicted in Figure 9 for the combined rankings from the three ‘agronomic’ datasets (‘full data’, Cont_SD1, and Cont_RF; Figure 9a), as well as for the rankings from the MP*N data (Figure 9b). Owing to several upper outliers, values greater than 15 were colored with the same blue shading to allow for better comparisons of the other values. Only rankings for trait*index combinations that reached R^2^ values > 0.20 are shown. For the following traits, no index exceeded this threshold for the agronomic datasets: NC of spikes at anthesis as well as the derived N traits PANup, CPostNup, and NupEff at anthesis. The rankings for the NC traits were diffuse with several indices reaching high rank values for each few traits, but on a low absolute R^2^ level (Figure 4). For most Nup traits, a clear advantage of most RE-based indices is visible. The indices NDRE_770_750, R780_R740, REIP, R760_R730, Maccioni, and RVSI excelled through high-rank sums for most of the Nup traits in both rankings. R787_765 and TCARI_OSAVI reached higher rank sums (WMMRS >17) for maturity NC and Nup traits as well as NTEff and NHI in the agronomic ranking, but not in the MP*N ranking. In contrast, most NIR/VIS and VIS indices were not particularly useful for the assessment of any N trait. Overall, only a few indices reached ‘relevant’ relationships (R^2^ > 0.20) in both rankings for most NC traits and derived N traits. This was also the case for direct Nup traits in the agronomic approach, while index rankings differed less in the MP*N approach.

To compare the possibility of transferring the optimized index selection between datasets, the rankings of both matrices were correlated against each other (Table 4; Figure S6). Interestingly, rankings were substantially more stable (Spearman’s ρ > 0.84 ***) for all traits of anthesis Nup, GNup (Nup Mat. grain), and total Nup at both growth stages, whereas rankings for leaf Nup at maturity were negatively correlated except for ‘other leaves.’ Rankings also differed substantially for most NC traits but were stable for the NC of grains (ρ = 0.91 ***) as well as of flag leaf–1 at anthesis. Among the other traits, stable rankings (ρ > 0.86 ***) were found for NT only.

### 3.4. Re-Assessing the Index Grouping

**Figure 10:**
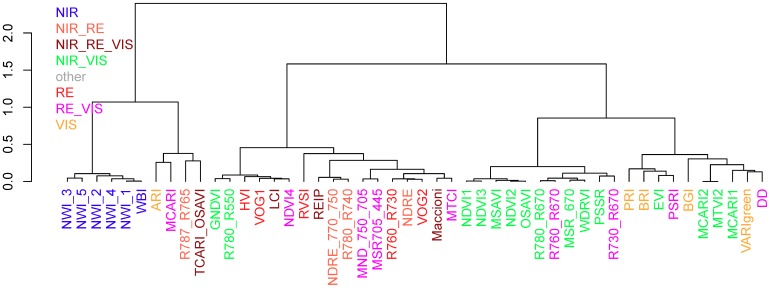
Cluster analysis based on Ward’s method on index kinship based on the performance for all dates and traits in the full data (*n* = 10*34 -> 340). Colors indicate initial index groups based on the included spectral regions (Figure 1b).

A cluster analysis was conducted based on the coefficients of determination for all trait*index*date combinations for comparing the initial index grouping according to the spectral regions with the grouping on the basis of the effective performance for all considered plant traits. For the full data, the clustering grouped the indices into two first-order branches. The NIR-based water band indices formed the most homogeneous group with a uniform second-order branch in the full data (Figure 10). The VIS indices were grouped in the second branch within the same first-order branch as the NIR indices, together with TCARI_OSAVI, R787_765, MCARI, DD, and PSRI as well as the NIR/VIS indices EVI, MCARI1, MCARI2, and MTVI2. The second first-order branch was subdivided into two branches as well: One dominated by most NIR/VIS indices, in addition to the R730_R670 and R760_R670, and the other branch containing most red edge-based indices as well as the NIR/green indices GNDVI and R780_R550. The grouping into four main groups was similar in all four datasets (not shown). In addition to the consistent groupings of the water-band indices, some indices were consistently grouped closely together: PRI + BRI, MCARI2 + MTVI2, TCARI_OSAVI + R787_R765, NDRE_770_750 + REIP + R780_R740, GNDVI + R780_R550, MND_750_705 + MSR705_445, R760_R730 + NDRE + VOG2 as well as HVI + VOG1+ LCI + NDVI4.

## 4. Discussion

This study evaluated the influence of the selection of measurement stages/dates and spectral vegetation indices (SVIs) on the estimation of various GNup-related N traits.

### 4.1. In-Season Estimation of Grain N Uptake and Contributing N Traits

Similar to grain yield (GY), GNup is formed over time but relies substantially more on the pre-anthesis component—Nup until anthesis—and its efficient translocation (NTEff). The resulting N translocation (NT) contributed on average ≈96% to GNup in RF and ≈91% in Cont and SD1, respectively, in the present data [12]. Although GNup was reduced by ≈9% in RF and increased by 5% in SD1, compared with Cont, NTEff (−8%), and NT (−5%, not significant) which were affected only in RF. The N harvest index (NHI) varied little between cultivars but was substantially higher than the DM harvest index (NHI ≈0.78 in RF, 0.84 in Cont and 0.85 in SD1). However, total Nup differed substantially both in the ‘agronomic’ datasets and between cultivars within the MP*N combinations. The fact that GNup was not closely related to GNC (R^2^ = 0.38 ***) in the full data [12] explains that the seasonal R^2^ patterns and the index rankings of GNup and GNC differed substantially. However, considering the high contribution of NT to GNup [12], high R^2^ values were reached for GNup already at stem elongation, the phase of maximum Nup [70]. The ear emergence stage was not suitable for detecting GNup and total Nup, which is likely an effect of phenological shifts that impact the spectral signal [71]. Although the seasonal pattern is consistent with previous years in the same environment [26], other studies recommend ear emergence for wheat [28] or anthesis for spring barley [72], indicating that the variation in ear emergence should be accounted for, depending on the underlying treatments.

Both the similar temporal R^2^ patterns and index rankings for total Nup as for GNup are in accordance with the close relationship between both traits (R^2^ = 0.88 *** in the full data and R^2^ = 0.64–0.97 within the MP*N datasets; [12]), whereas NHI was poorly (R^2^ = 0.17 * in the full data) or not (in MP*N) related to GNup. The estimation of NHI—only driven by its lower values in RF compared with Cont and SD1—may relate to the detection of differences in leaf senescence. Testing the spectral estimation of various biomass and N traits for phenotyping breeding lines, Frels et al. found useful relationships for NHI only in one of two years from the TCARI_OSAVI. In contrast to the present study, they also reported moderate, yet year-dependent, correlations for PANup; however, for a substantially broader data range in both traits.

The clear advantage of most red edge (RE)-based indices for most Nup traits is well in line with previous findings regarding the higher sensitivity of the RE reflection for overcoming saturation in the red region and for estimating the N status [27,54,73,74,75]. Thus, in particular NIR/RE indices that replaced the VIS band with a RE band [76] performed well. In contrast, the placement of the right-sided band appeared to be less crucial as indicated by the similarity in rankings from the R760_R730, R780_R740, NDRE, and NDRE_770_750 as well as of the LCI and the Maccioni index. Thus, the Maccioni index was previously recommended for GNup and NupEff [28], the NDRE_770_750 [26] and the REIP [77] for GNup, and the R780_740 for total Nup and for N use efficiency [27]. In contrast, some RE/VIS indices—notably the NDVI4, MTCI, MSR_705_445 and MND_750_705—ranked similarly to each other, but rarely best for any of the traits.

The comparable seasonal R^2^ pattern and index rankings for GNup as for total Nup at anthesis relates to the correlations between the traits (r = 0.48 ***; not shown). However, GNup was better assessed than vegetative Nup, possibly owing to the stage-specific sampling at anthesis, including a sampling interval of a few days, which reduced the variance in vegetative Nup. The mostly better detection of Nup of leaves than that of stems and spikes/chaff confirms results on spring barley [18] and is plausible, considering that the sensor predominantly detects the horizontally aligned leaf blades [78]. Thus, the observed detection of other organs may indicate rather indirect relationships via correlations with leaf traits. In contrast to anthesis Nup, vegetative Nup at maturity was substantially influenced by the reduced NTEff as introduced by reduced fungicide, which correlated with total leaf Nup at maturity (full data: r = 0.79 ***; not shown). Thus, indices suited for NTEff as well as for NC (R787_R765 and TCARI_OSAVI) were able to detect vegetative Nup at maturity in the agronomic data (Figure 7), but not the cultivar differences in MP*N. The similarly high rankings of both indices for many organ-level NC and Nup traits at maturity, NTEff, and NHI suggest a specific senescence sensitivity of these indices, because all these traits were altered by fungicide and N level effects [12]. This is in line with previous recommendations of these indices for retrieving NC in grassland [52] or chlorophyll [55], respectively.

### 4.2. Estimation of N Concentration Traits

The estimation of NC or chlorophyll content previously focused on leaf NC or NC of the total plant for optimizing N fertilization before anthesis [15]. However, the resulting N status at anthesis and grain filling is crucial for the formation of GY and GNup as well. Although RE/VIS indices previously optimized for leaf NC or chlorophyll content including the DD [60], MND_750_705, MSR_705_445 [62], and MTCI [63], ranked high for either one of the leaf layers, the VIS indices blue/red pigment index (BRI) and BGI were more consistent over time, for all three leaf layers and both in the agronomic and the MP*N approaches. Blue bands were less commonly used, but the BGI was also confirmed for chlorophyll retrieval in barley [79] and vine [48], in contrast to the BRI which was found less useful [48]. Such VIS indices are interesting because they are also available from simple red-green-blue (RGB) cameras [80]. Similar to Nup, NC of vegetative organs at maturity relates to variation in NTEff. Thus, the fungicide-related variation in the full data was captured by indices sensitive to senescence, whereas in the MP*N data, cultivars may not have sufficiently differed in NTEff.

### 4.3. Stability of Index Rankings by Datasets and Index Grouping

**Figure 11:**
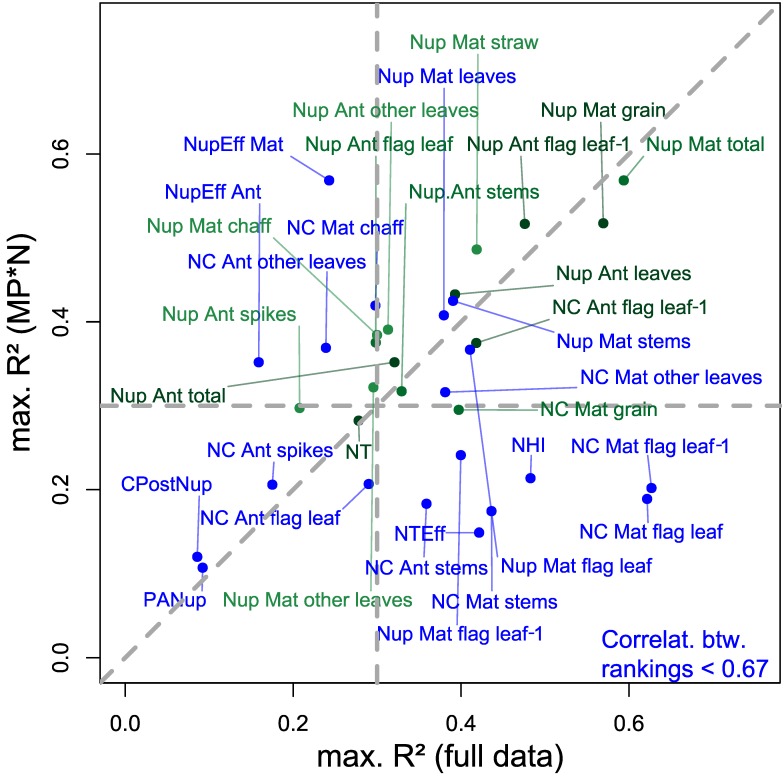
Relationship of trait-specific maximum relationships (R^2^; Figure 4) in the full data and the MP*N approach. The data is colored by Spearman’s ρ (green: ρ > 0.67, blue: ρ < 0.67) on the correlations between the WMMRS rankings of the ‘agronomic’ and the MP*N approach (Table 4). Dashed lines mark the 1:1 line and arbitrary R^2^ thresholds (0.3) for categorizing the traits by R^2^ values in both approaches.

Year and treatment effects can be substantial, particularly on the derived N traits [20]; therefore, the comparison of index rankings addresses the influence of the underlying treatments. Similarly as for most Nup traits, the constant index rankings found for the important traits GNup and GNC are promising (Table 4; Figure 11). In contrast, trait variation introduced by N fertilization and differing fungicide intensities in the agronomic data may explain substantially differing index rankings (correlations between rankings < 0.67; blue colored in Figure 11) for many maturity-related NC and Nup traits. Mostly, those traits were not reliably assessed in MP*N but they were in the full data approach (bottom right quadrant in Figure 11). While indices selected for green-colored traits (transferable rankings) are promising for a more independent application, the index selection for blue-colored traits would require more consideration of the underlying treatments and/or trial environments.

The SVI clustering confirms the similarity in the index rankings and indicates that most SVIs grouped together by spectral bands gather similar canopy information. This is particularly the case for the NIR-based indices, which include the same water absorption band, as well as for the VIS indices, whereas the delimitation of RE-SVIs was less stringent. The similar grouping of the R787_R765 and the TCARI_OSAVI with VIS indices is in line with their discussed sensitivity of senescence-related pigment information. Similarly, RE/VIS indices, which were previously optimized for pigment detection (DD, MCARI, and PSRI [43,61,62]), were grouped together with VIS indices. Some RE/VIS indices based on the R670 band being grouped with the NIR/VIS indices support that choosing an upper band left to the NIR plateau had little impact compared to NIR/VIS indices similar to the NDVI.

The hyperspectral data used in this study yields optimized narrow-band SVIs. The information of relevant spectral bands used by these SVIs can be transferred to the band selection and the design of optimum multispectral sensors. Such sensors are less expensive, more robust and therefore better suited to be used by practitioners on tractors or drone platforms. Further aspects of view geometry, sensor resolution, and illumination conditions need to be addressed in this context.

## 5. Conclusions

Aiming at exploiting spectral proximal sensing for practical use in agronomic field trials, optimizations are required in the selection of (i) vegetation indices depending on the sensor and (ii) suitable growth stages for measurements, while a better understanding of the traits contributing to final GNup and their treatment response is equally important [12] for understanding (iii) the trait detection. With respect to (i), the results confirm the usefulness of NIR/red edge indices. For (ii), although the milk ripeness stage was generally best suited, moderate relationships observed already at stem elongation have the advantage of seasonally earlier estimations. In contrast, the ear emergence/anthesis phase should be avoided, probably owing to phenological shifts among cultivars. With respect to (iii), the results suggest that GNup is well estimated spectrally, similarly to total Nup under various trial conditions, whereas partitioning to the grain is not generally visible to the sensor. Although leaf information was better detected, N status of other organs could be moderately estimated as well, however the mechanism of the organ-specific detection may be indirect and should be further evaluated. The N translocation could be estimated in a similar manner as the closely related Nup at anthesis. Several traits—including most vegetative NC and Nup traits at maturity, NHI, and NTEff—were detected in the context of accelerated senescence without leaf fungicide in RF, but not for genotypic variation in MP*N.

## Figures and Tables

**Table 1:** Dates of spectral measurements (month/day) with days after sowing (d.a.s) in control (Cont) and reduced fungicide (RF) plots and dominant growth stages.

Date (Month/Day)	d.a.s.	Growth Stage
03/31	159	leaf development
04/13	172	tillering
05/17	206	stem elongation
05/25	214	booting
06/01	221	ear emergence
06/08	228	anthesis
06/21	241	early milk
06/26	246	late milk
07/01	251	early dough
07/04	255	soft dough

**Table 2:** List of spectral vegetation indices considered in this study. ‘R’ denotes the reflection in indicated wavebands.

Index	Full name	Spectral	Equation	Reference
NWI-1	Normalized water index 1	NIR	(R970−R900)/(R970+R900)	[31,32]
NWI-2	Normalized water index 2	NIR	(R970−R850)/(R970+R850)	[31,32]
NWI-3*	Normalized water index 3	NIR	(R970−R920)/(R970+R920)	[31]
NWI-4*	Normalized water index 4	NIR	(R970−R880)/(R970+R880)	[31]
NWI-5	Normalized water index 5	NIR	(R970−R930)/(R970+R930)	[26]
WBI	Water band index	NIR	R900/R970	[33]
EVI	Enhanced vegetation index	NIR,VIS	$2.5*\frac{\left(R864-R670\right)}{\left(R864+6*R670-7.5*R420+1\right)}$	[34]
GNDVI	Green NDVI**	NIR,VIS	(R780−R550)/(R780+R550)	[35]
MCARI1	Modified chlorophyll absorption in reflectance index 1	NIR,VIS	1.2∗(2.5∗(R800−R670)−1.3∗(R800−R550))	[36]
MCARI2	Modified chlorophyll absorption in reflectance index 2	NIR,VIS	1.5∗(2.5∗(R800−R670)−1.3∗(R800−R550))/ (((2*$\;R800+1){}^{2}-\left(6*R800-5*R670^{0.5}\right)-0.5{)}^{0.5})$	[36]
MSAVI	Modified soil-adjusted vegetation index	NIR,VIS	$(2*R800+1-((\left(2*R800+1\right){}^{2}-$ $8*\left(R800-R670\right){)}^{0.5}))/2$	[37]
MSR(MSR_670)	Modified simple ratio 670	NIR,VIS	$\frac{\left(R800/R670-1\right)}{\left({\left(R800/R670+1\right)}^{0.5}\right)}$	[38]
MTVI2	Modified triangular vegetation Index 2	NIR,VIS	1.5(1.2∗(R800−R550)−2.5∗(R670−R550))/ $({\left({\left(2*R800+1\right)}^{2}-\left(6*R800-5*{\left(R670\right)}^{0.5}-0.5\right)\right)}^{0.5}$)	[36]
NDVI1	Normalized difference vegetation index 1	NIR,VIS	(R864−R670)/(R864+R670)	[39]
NDVI2	Normalized difference vegetation index 2	NIR,VIS	(R780−R670)/(R780+670)	[19,40]
NDVI3	Normalized difference vegetation index 3	NIR,VIS	(R900−R670)/(R900+R670)	[41]
OSAVI	Optimized soil-adjusted vegetation index	NIR,VIS	$\frac{\left(1+0.16\right)*\left(R800-R670\right)}{\left(R800+R670+0.16\right)}$	[42]
PSSR	Pigment specific simple ratio	NIR,VIS	R800/R500	[43]
R780_R550		NIR,VIS	R780/R550	[44]
R780_R670		NIR,VIS	R780/R670	[45]
WDRVI	Wide dynamic range vegetation index	NIR,VIS	$\frac{\left(0.1*R780-R670\right)}{\left(0.1*R780+R670\right)}$	[46]
ARI	Anthocyanin reflectance index	VIS	1/R550−1/R700	[47]
BGI	Blue green pigment index	VIS	R450/R550	[48]
BRI	Blue red pigment index	VIS	R450/R690	[48]
PRI	Photochemical reflectance index	VIS	(R531−R570)/(R531+R570)	[49]
VARIgreen	Visible atmospherically resistant vegetation index green	VIS	(R550−R670)/(R550+R670−R470)	[50]
NDRE	Normalized difference NIR/red edge index	NIR,RE	(R790−R720)/(R790+R720)	[51]
R780_R740		NIR,RE	R780/R740	[29]
NDRE_770_750		NIR,RE	(R770−R750)/(R770+R750)	[26]
R787_R765		NIR,RE	R787/R765	[52]
LCI	Leaf chlorophyll index	NIR,RE,VIS	(R850−R710)/(R850+R680)	***
Maccioni	Maccioni index	NIR,RE,VIS	(R780−R710)/(R780−R680)	[53]
REIP	Red edge inflection point	NIR,RE,VIS	$700+40*\frac{\left(\frac{R670+R780}{2}\right)-R700}{\left(R740-R700\right)}$	[54]
TCARI_OSAVI		NIR,RE,VIS	3*((R700-R670)-0.2*(R700-R550)* R700/R670)/ (1+0.16)∗(R800−R670)/(R800+R670+0.16)	[55]
HVI	Hyperspectral vegetation index	RE	R750/R700	[56]
R760_R730		RE	R760/R730	[30,57]
RVSI	Red edge vegetation stress index	RE	(R714+R752)/2−R733	[58]
VOG1	Vogelmann 1	RE	R740/R720	[59]
VOG2	Vogelmann 2	RE	(R734−R747)/(R715+R726)	[59]
DD	Double difference index	RE,VIS	(R749−R720)−(R701−R672)	[60]
MCARI	Modified chlorophyll absorption in reflectance index	RE,VIS	((R700−R670)−0.2∗(R700−R550))∗(R700/R670)	[61]
MND_750_705	Modified normalized difference 750/705	RE,VIS	$\frac{\left(R750-R705\right)}{\left(R750+R705-2*R445\right)}$	[62]
MSR_705_445	Modified simple ratio 705/445	RE,VIS	(R750−R445)/(R705−R445)	[62]
MTCI	MERIS**** terrestrial chlorophyll index	RE,VIS	(R750−R710)/(R710−R680)	[63]
NDVI4	Normalized difference vegetation index 4	RE,VIS	(R750−R705)/(R750+R705)	[64]
PSRI	Plant senescence reflectance index	RE,VIS	(R680−R500)/R750	[62]
R730_R670		RE,VIS	R730/R670	[30]
R760_R670		RE,VIS	R760/R670	[65]

* The equations of NWI−3 and NWI−4 are used as in the original publication [31] whereas the names are interchanged amongst others in [66,67,68,69]; ** Normalized difference vegetation index; *** www.indexdatabase.de; **** MEdium Resolution Imaging Spectrometer.

**Table 3:** Best spectral vegetation indices (SVIs) based on the weighted mean/maximum-rank sums (WMMRS) from the ‘agronomic’ approach with its seasonally highest R^2^ value with significance (*** *p* < 0.001; ** *p* < 0.01; * *p* < 0.05; n.s.: not significant) and lowest root mean square error (RMSE) reached on the optimum date (month/day) in the different datasets: ‘Full data’, ‘Cont_SD1’, ‘Cont_RF’ as well as the six main plot*N level combinations (MP*N). Considering the six MP*N blocks as different environments, the results for MP*N are based on the average R^2^ and RMSE values from the six MP*N subsets. Due to slightly differing *n* (number of data points), the significance for MP*N was re-calculated based on the averaged thresholds in the six subsets. For nitrogen uptake (Nup), ‘Ant. Leaves’ and ‘Mat. Leaves’ refer to the aggregated values of all leaf layers. Abbreviations: NC: nitrogen content; Nup: nitrogen uptake; Ant.: anthesis; Mat.: maturity; PANup: post-anthesis N uptake; CPostNup: contribution of PANup to total Nup; NT: N translocation; NTEff: N matter translocation efficiency; NHI: N harvest index. Refer to Figure 5, Figure 7 and Figure 8 for seasonal R^2^-values of selected traits in the full data and MP*N.

				Seasonally Best R^2^								Optimum Date (month/day)				RMSE			
Trait Group	Trait	Best SVI	WMMRS	Full Data		Cont_SD1		Cont_RF		MP*N		Full Data	Cont_SD1	Cont_RF	MP*N	Full Data	Cont_SD1	Cont_RF	MP*N
NC (%)	Ant. spikes	NDRE_770_750	13	0.16	***	0.25	***	0.09	**	0.18	*	06/26	06/26	06/26	06/21	0.06	0.06	0.06	0.05
	Ant. stems	RVSI	19	0.34	***	0.44	***	0.30	***	0.12	n.s.	07/01	06/26	05/17	05/17	0.10	0.09	0.10	0.08
	Ant. flag leaf	DD	17	0.25	***	0.20	***	0.20	***	0.16	*	05/25	05/25	05/25	05/25	0.19	0.20	0.20	0.18
	Ant. flag leaf–1	BGI	19	0.30	***	0.30	***	0.27	***	0.28	**	06/08	06/08	06/08	06/08	0.20	0.20	0.20	0.17
	Ant. other leaves	BGI	36	0.24	***	0.27	***	0.25	***	0.29	**	06/08	06/08	07/01	06/08	0.24	0.23	0.24	0.21
	Mat. grain	REIP	17	0.38	***	0.41	***	0.41	***	0.26	**	05/25	06/26	05/25	05/25	0.14	0.14	0.13	0.09
	Mat. chaff	TCARI_OSAVI	18	0.30	***	0.21	***	0.28	***	0.17	*	07/04	07/04	06/26	06/08	0.05	0.05	0.06	0.05
	Mat. stems	TCARI_OSAVI	17	0.44	***	0.36	***	0.38	***	0.17	*	07/04	07/01	06/26	07/01	0.04	0.04	0.04	0.03
	Mat. flag leaf	R787_R765	16	0.62	***	0.08	*	0.69	***	0.14	*	07/04	06/26	07/04	03/31	0.26	0.11	0.26	0.16
	Mat. flag leaf–1	MCARI	17	0.49	***	0.18	***	0.59	***	0.09	n.s.	07/04	07/01	07/04	07/01	0.16	0.08	0.16	0.09
	Mat. other leaves	MCARI	14	0.37	***	0.15	***	0.52	***	0.13	n.s.	07/01	06/08	07/01	07/01	0.13	0.11	0.13	0.10
Direct Nup (kg N ha^−1^)	Ant. spikes	BRI	13	0.21	***	0.32	***	0.19	***	0.28	**	06/26	07/01	06/26	06/26	5.2	4.5	5.4	4.8
	Ant. stems	NDRE_770_750	15	0.33	***	0.36	***	0.33	***	0.28	**	05/25	05/25	04/13	05/25	10.3	9.3	10.3	9.4
	Ant. flag leaf	NDRE_770_750	13	0.30	***	0.43	***	0.22	***	0.33	***	07/04	07/04	07/04	07/04	5.6	4.8	6.2	4.9
	Ant. flag leaf–1	REIP	14	0.40	***	0.48	***	0.43	***	0.51	***	05/25	06/21	06/08	06/08	3.8	3.5	3.5	3.1
	Ant. other leaves	ARI	14	0.16	***	0.17	***	0.30	***	0.39	***	06/26	05/17	04/13	04/13	4.4	4.3	3.8	3.3
	Ant. leaves	NDRE_770_750	14	0.38	***	0.47	***	0.33	***	0.43	***	06/08	06/08	06/08	06/08	10.0	9.3	10.7	9.1
	Mat. grain	NDRE_770_750	15	0.57	***	0.53	***	0.67	***	0.52	***	06/26	06/26	06/26	06/26	16.0	16.7	13.1	9.7
	Mat. chaff	REIP	14	0.25	***	0.36	***	0.24	***	0.31	***	06/26	06/26	04/13	06/26	1.5	1.3	1.5	1.3
	Mat. stems	TCARI_OSAVI	15	0.39	***	0.27	***	0.46	***	0.18	*	06/26	07/01	06/26	07/01	3.1	2.7	3.0	2.6
	Mat. flag leaf	TCARI_OSAVI	19	0.39	***	0.31	***	0.43	***	0.27	**	07/04	07/04	07/04	07/01	2.2	0.8	2.4	1.2
	Mat. flag leaf–1	TCARI_OSAVI	19	0.40	***	0.22	***	0.46	***	0.14	*	07/04	07/01	07/04	07/01	0.8	0.5	0.8	0.5
	Mat. other leaves	R787_R765	19	0.30	***	0.50	***	0.30	***	0.32	***	05/25	06/08	07/04	06/08	0.9	0.7	0.9	0.8
	Mat. leaves	TCARI_OSAVI	20	0.37	***	0.22	***	0.46	***	0.23	**	07/04	06/26	07/04	07/01	3.6	1.9	3.7	2.1
	Ant. total	NDRE_770_750	14	0.32	***	0.43	***	0.27	***	0.34	***	05/25	06/08	06/08	06/08	23.4	20.6	24.4	21.3
	Mat. total	NDRE_770_750	16	0.59	***	0.57	***	0.58	***	0.57	***	06/26	06/26	06/26	06/26	17.5	19.0	16.9	11.7
	Mat. straw	TCARI_OSAVI	18	0.42	***	0.27	***	0.51	***	0.22	**	06/26	06/26	06/26	04/13	6.9	5.2	6.6	5.0
Derived N	PANup (kg N ha^−1^)	RVSI	17	0.09	***	0.13	***	0.11	**	0.10	n.s.	06/26	05/17	06/26	07/04	23.9	22.4	24.1	19.6
	CPostNup	RVSI	16	0.07	**	0.11	**	0.09	**	0.10	n.s.	04/13	07/04	06/26	07/04	0.11	0.10	0.11	0.09
	NT (kg N ha^−1^)	NDRE_770_750	13	0.28	***	0.33	***	0.26	***	0.28	**	06/08	06/08	06/08	06/08	21.1	19.2	21.7	19.7
	NTEff	R787_R765	20	0.42	***	0.07	*	0.54	***	0.08	n.s.	07/04	05/17	07/04	03/31	0.03	0.02	0.03	0.02
	NHI	NWI_2	15	0.32	***	0.10	**	0.46	***	0.13	n.s.	07/04	06/08	07/04	06/01	0.03	0.02	0.03	0.02
	NupEff Ant.	RVSI	28	0.16	***	0.23	***	0.14	***	0.33	***	06/26	07/04	06/26	06/08	0.72	0.65	0.75	0.25
	NupEff Mat.	R787_R765	32	0.24	***	0.49	***	0.16	***	0.33	***	06/26	06/26	06/26	05/25	0.59	0.47	0.62	0.18

**Table 4:** Spearman’s ρ and significance level (*** *p* < 0.001; ** *p* < 0.01; * *p* < 0.05; n.s.: not significant) of the correlations between the agronomic and the MP*N index rankings (Figure 9) by target trait. See Figure S6 for scatterplots between both rankings.

NC			Nup			Derived N		
Ant. spikes	−0.29	*	Ant. spikes	0.85	***	PANup	0.06	n.s.
Ant. stems	0.02	n.s.	Ant. stems	0.89	***	CPostNup	−0.03	n.s.
Ant. flag leaf	0.30	*	Ant. flag leaf	0.90	***	NT	0.97	***
Ant. flag leaf–1	0.92	***	Ant. flag leaf–1	0.92	***	NTEff	−0.22	n.s.
Ant. other leaves	0.45	**	Ant. other leaves	0.84	***	NHI	−0.37	*
Mat. grain	0.91	***	Ant. leaves	0.98	***	NupEff Ant.	0.35	*
Mat. chaff	0.54	***	Mat. grain	0.94	***	NupEff Mat.	0.17	n.s.
Mat. stems	−0.12	n.s.	Mat. chaff	0.78	***			
Mat. flag leaf	−0.03	n.s.	Mat. stems	0.60	***			
Mat. flag leaf–1	0.09	n.s.	Mat. flag leaf	−0.34	*			
Mat. other leaves	0.58	***	Mat. flag leaf–1	−0.53	***			
			Mat. other leaves	0.80	***			
			Mat. leaves	−0.33	*			
			Ant. total	0.95	***			
			Mat. total	0.89	***			
			Mat. straw	0.63	***			

## Supplementary Materials

The following are available online at https://www.mdpi.com/1424-8220/19/21/4640/s1. Table S1: List of plant traits considered in this study, grouped by trait groups. Figure S1: Field trial measurements on 21 June 2017, colored by the values of the simple ratio index R760/R730. Figure S2: Plot-level spectra acquired on 31 March (leaf development, left), 17 May (stem elongation; middle) and 4 July 2017 (soft dough; right). Figure S3: The influence of the measurement date (year/month/day) on the trait estimation (Cont_SD1/Cont_RF/averaged form the six MP*N datasets). Figure S4: The index suitability by target trait. Figure S5: Seasonal coefficients of determination (R^2^) reached from all indices for grain N uptake (GNup), total Nup at maturity, and the N harvest index (NHI) for the Cont_SD1 and the Cont_RF data. Figure S6: Consistency between WMMRS-index rankings from the MP*N datasets (y-axis; Figure Figure 9b) and the agronomic datasets (x-axis; Figure Figure 9a) for all considered plant traits.
