# Supplementary material for: Temporal and Spectral Optimization of Vegetation Indices for Estimating Grain Nitrogen Uptake and Late-Seasonal Nitrogen Traits in Wheat
Source: Sensors (Basel). 2019 Oct 25;19(21):4640. doi: 10.3390/s19214640 (PMC6864866; doi:10.3390/s19214640)
Supplement: Supplementary file 1 [file sensors-19-04640-s001.pdf]

sensors

Supplementary Material

Table S1: List of plant traits considered in this study, grouped by trait groups.

Trait group	Plant trait	Abbreviation	Description
N concentration traits [%]	organ-specific N concentration (vegetative)	NC [organ-name]	at anthesis and maturity for flag leaf, flag leaf-1, ‘other leaves’ and culms, at anthesis for spikes and at maturity for chaff
	grain N concentration	GNC	
(direct) N uptake traits [kg ha^{-1}]	organ-specific N uptake (vegetative)	Nup [organ-name]	at anthesis and maturity for flag leaf, flag leaf-1, ‘other leaves’, culms, and summed up for the three leaf layers at anthesis for spikes and at maturity for chaff
	total N uptake	Nup_{Ant}, Nup_{Mat}	sum of organ-specific DM-traits at anthesis and maturity
	N uptake straw	Nup straw	sum of organ-specific vegetative N uptake at maturity
	grain N uptake	GNup	
derived N traits	post-anthesis N uptake	PANup	
	contribution of post-anthesis nitrogen to total nitrogen uptake	CPostNup	
	total N translocation	NT	
	N translocation efficiency	NTEff	
	N harvest index	NHI	relative N partitioning to the grain
	apparent N uptake efficiency at anthesis	NupEff_Ant.	
	apparent N uptake efficiency at maturity	NupEff Mat.	

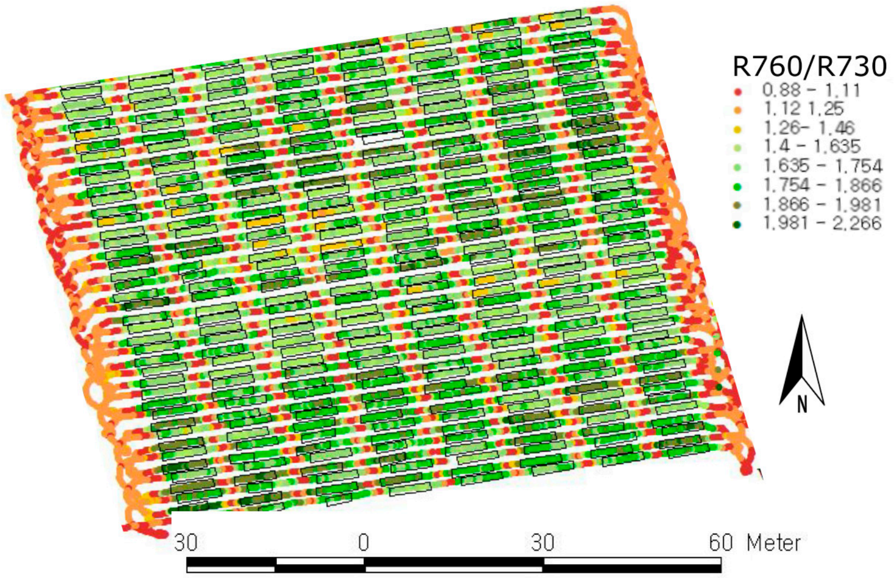

Figure S1: Field trial measurements on 21 June 2017, colored by the values of the simple ratio index R760/R730. Colored points indicate individual measurements, captured at a frequency of 5 Hz. Red points indicate non-vegetated soil strips between plots. Polygons delimit the data extracted on the plot level.

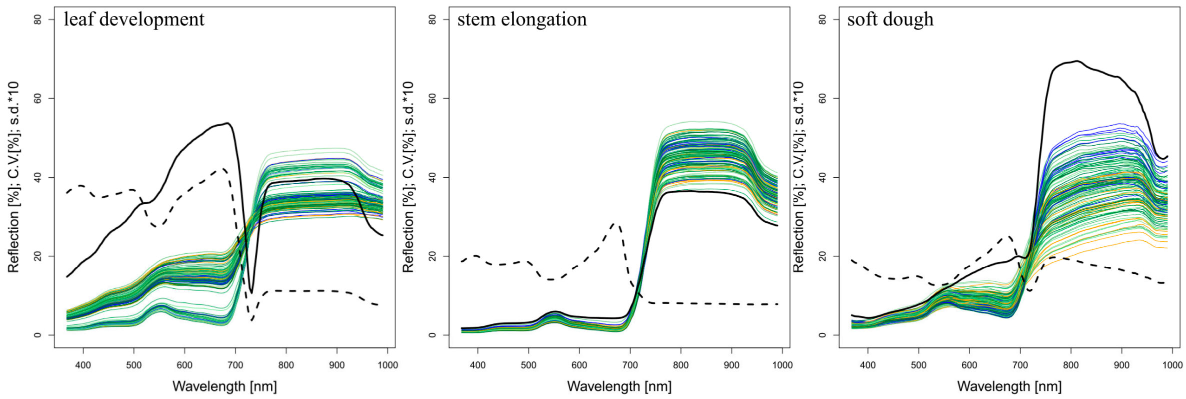

Figure S2: Plot-level spectra acquired on 31 March (leaf development, left), 17 May (stem elongation; middle) and 04 July 2017 (soft dough; right). The spectra are colored by grain N uptake (GNup; yellow: low GNup, blue: high GNup). The dashed line represents the coefficient of variation of the reflectance and the solid line the standard deviation multiplied by 10.

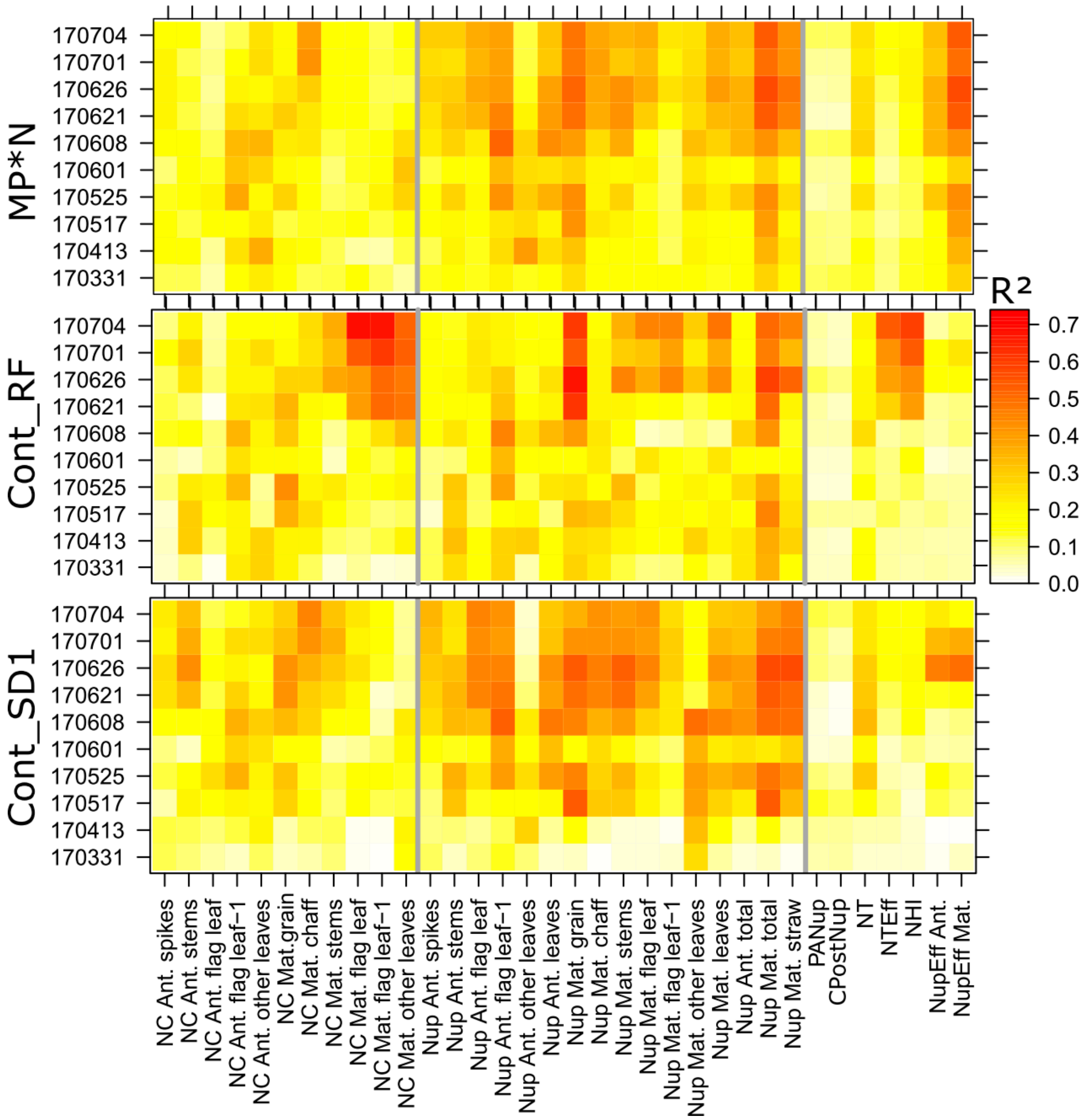

Figure S3: The influence of the measurement date (year/month/day) on the trait estimation (Cont_SD1/Cont_RF/averaged form the six MP*N datasets): Maximum coefficients of determination (R^2) found from the 48 tested SVIs. Gray lines delimit trait groups.

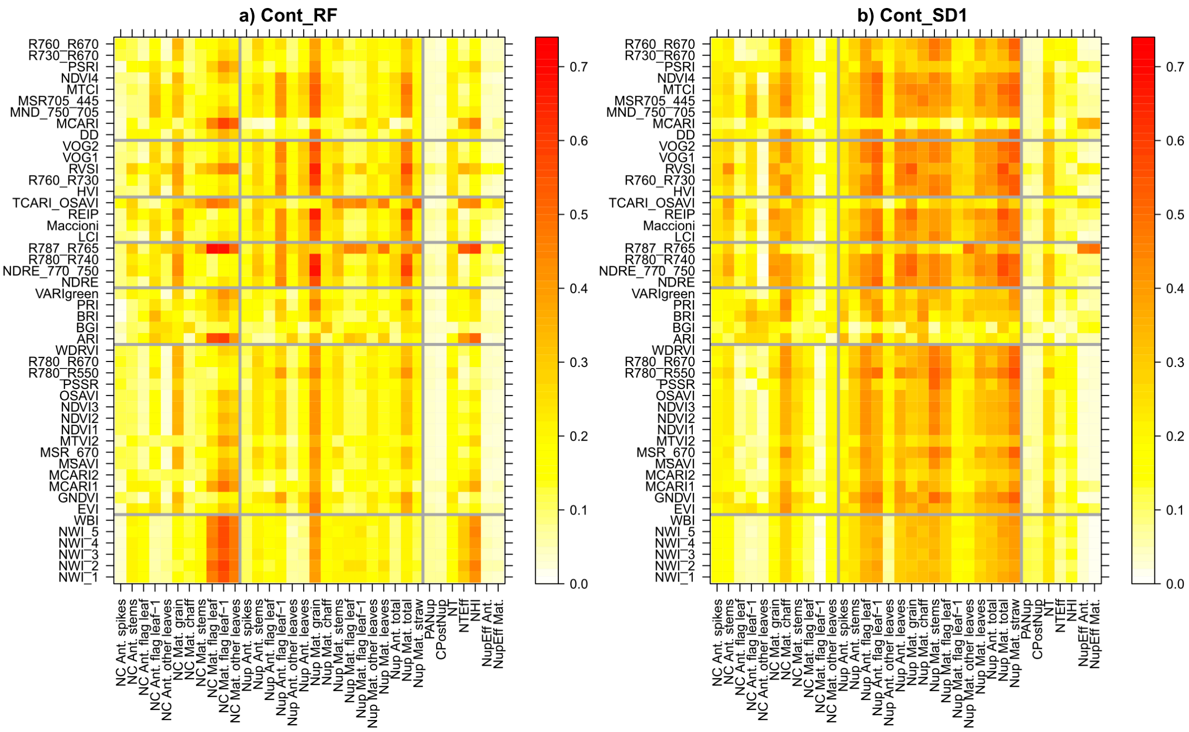

Figure S4: The index suitability by target trait (Cont_SD1/Cont_RF): Maximum coefficients of determination (R^2) found for each index*trait combination from 11 measurement dates. Gray lines delimit index and trait groups.

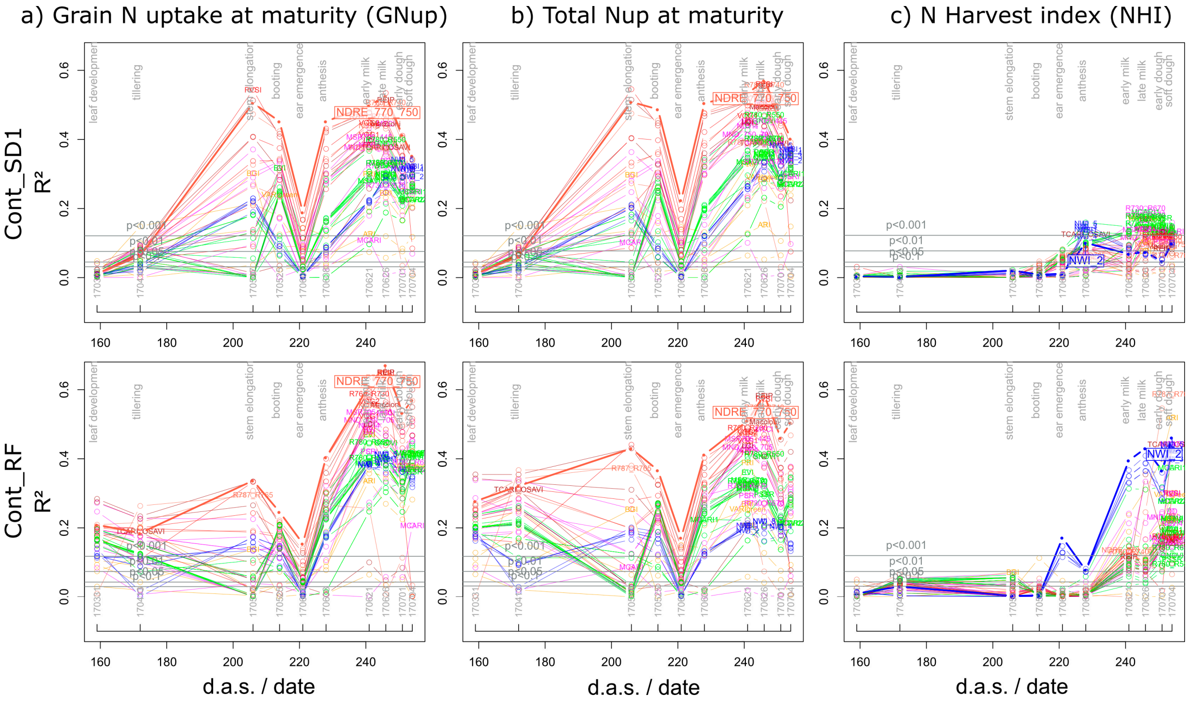

Figure S5: Seasonal coefficients of determination (R^2) reached from all indices for grain N uptake (GNup), total Nup at maturity, and the N harvest index (NHI) for the Cont_SD1 and the CONT_RF data. Refer to Figure 5 for results of the MP*N and the full data**.** Lines are colored according to the spectral regions included in the SVI equations (Figure 1). Horizontal gray lines indicate significance thresholds on different levels.

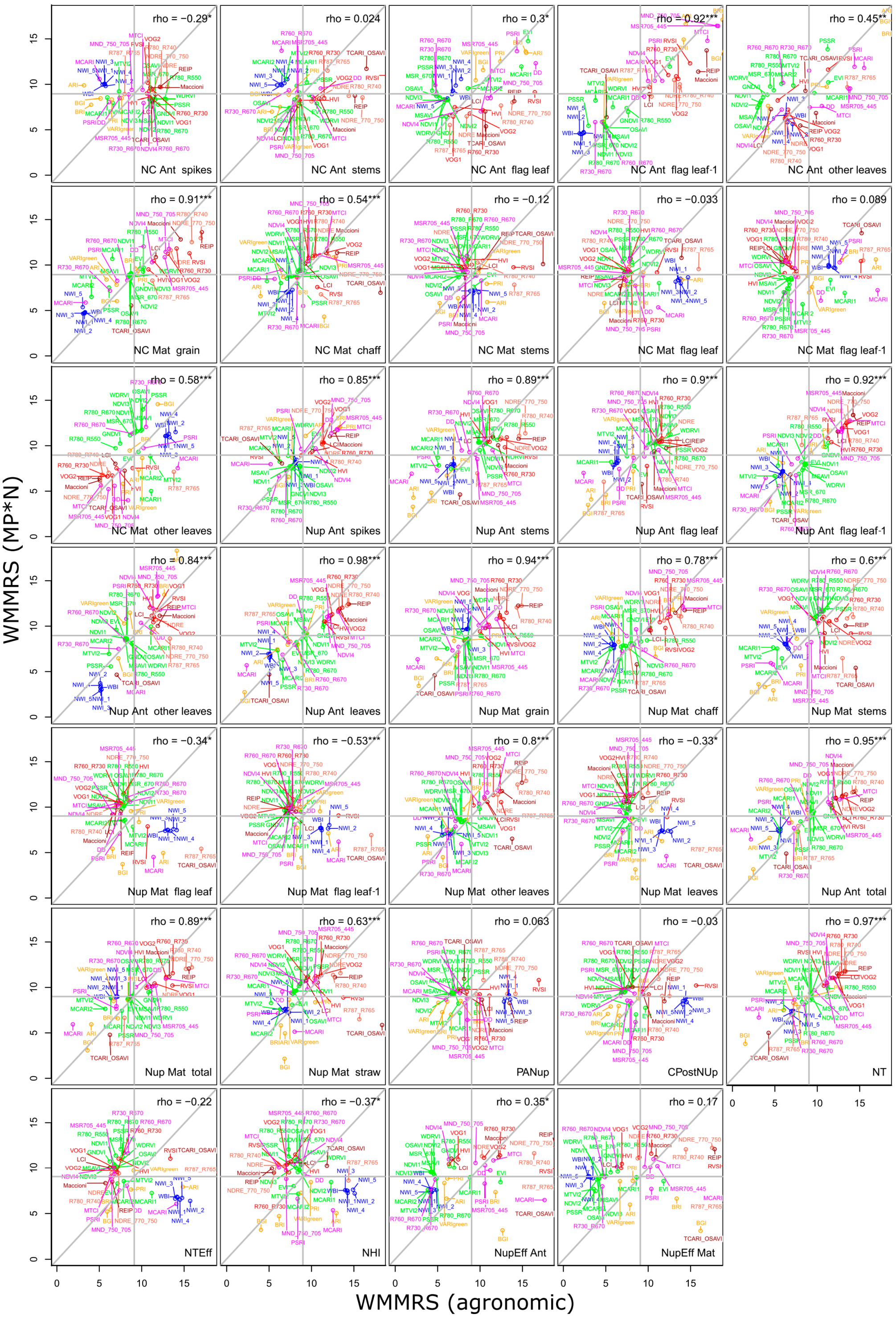

Figure S6: Consistency between WMMRS-index rankings from the MP*N datasets (y-axis; Figure 9 b) and the agronomic datasets (x-axis; Figure 9a) for all considered plant traits. The closer the correlation (Spearman's rho), the better the transferability of index selections between datasets. Gray lines indicate average rankings of 9. X- and y-axes span form 0 to 18. Rankings > 18 were neglected.
